# Successful Operation on, and Treatment of, a Disease of Antrum Highmorianum of the Right Side

**Published:** 1851-01

**Authors:** Charles H. Dubs

**Affiliations:** Dentist, Natchez.


### ARTICLE VII.

*Successful Operation on, and Treatment of, a Disease of Antrum Highmorianum of the Right Side.* By CHARLES H. DUBS, Dentist, Natchez.

*Description.*—The patient, Dudley Hunt, in his fifteenth year of age, had been afflicted with a terrible disease for eight months without any proper surgical treatment, his parents being under the impression it was only the effect of a severe cold. He was first brought to me on the third of April, 1845, and on examination, I found there was considerable expansion of the bones of the cheek and nose of the right side. The palate bone protruded much into the mouth, and the extension of the bones were so great as to produce an entire obstruction of the right nostril, also the swelling so as to raise the floor of the orbit, pushing the eye so far out of its socket, as to cause total loss of vision on that side. There was a fistulous opening at the prominent part of the malar bone, of a fleshy nature, from which pus was issuing, and communicated with an opening in the gum opposite to the anterior bicuspid tooth of the right side. There was also another fistula near the canthus of the eye, opening into the right nasal cavity.

*Operation and treatment.*—The first and second superior molars being much decayed in the crown, I extracted them, and perforated through the socket into the cavity, causing considerable pain on the entrance of the trocar. A large quantity of greenish and yellow pus discharged freely from the opening and was assisted by several injections into the sinus, which our youthful sufferer bore admirably and the flow being very copious, in a little time afforded him material relief. After this a cool emollient poultice was applied over the whole surface of the affected part.

April 4th. Very little pain this morning, and the swelling somewhat subsided, but found the external openings, as also that in the mouth, suppurating profusely and emitting a very fetid odor. On this account, I injected the sinus with a weak solution of chloride of soda in the proportion of one to twelve of aqua rose, lukewarm. This gave very little pain, and was followed by much coagulated lymph, and hard curdled matter. The emollient poultice of slippery elm continued morning and evening.

April 5th. On examination, I found the parietes of the alveoli much decayed, and also the external plate of the jaw. My patient was so timid that he required much persuasion before he would submit to another operation. I now removed the whole decayed part of the upper maxillary, extending from the posterior bicuspis near the socket of the dens sapientiæ, and thus formed a proper outlet. Occasional injections into the sinus were followed by much lumpy and fetid matter, along with a number of fragments of exfoliated bone. The nasal opening being completely closed, the solution found its outlet through the fistulous opening near the internal canthus. On examining this cavity with a probe, I found a large elastic substance which I judged to be a fungous mass. This the patient refused to allow me to remove, so that I had to confine myself to the use of injections, of a weak solution of chloride of soda, tannin and myrrh, until the 8th of April, when he consented to submit to the operation. Though the parts were very much inflamed and painful, I cut the fungus entirely away into two pieces, which together were about the size of an egg. Much lumpy matter and small pieces of dead bone followed. The injections and poultices continued.

April 9th. Removed a piece of exfoliated bone from the fistula near the canthus, it being a portion of the nasal bone. I also discovered the palatal bones in a state of necrosis, emollients continued.

April 10th. I made an incision from one fistula in the cheek, to the other in form of an angle, which enabled me to remove several pieces of bone that were exfoliated, and among them was a part of the malar and nasal bones. I now gave injections of sulphate of zinc, dissolved in warm water, thrown into the opening, and the lotion of chloride of soda into the sinus, and was much pleased to find the nasal opening pervious.

April 14th. The diseased parts have been discharging freely, and the patient doing remarkably well up to this time.

April 17th. Removed more loose bone from the opening in the cheek, the suppuration is decreased, the patient is recovering his spirits, and the sight of the eye has much improved. For a great number of red spots on the face I prescribed a bottle of Sands' sarsaparilla, injections and emollients continued.

April 23d. The diseased parts continued to improve, and the red spots on the face have entirely disappeared.

April 25th, 28th and 30th. Removed more dead bone from the superior maxillary, and palatal bones, usual injections, &c., continued.

May 2d and 7th. The health of the patient, and diseased parts still improving, the latter suppurating freely. On examining the lower jaw of the left side I discovered a hard tumor directly below the first inferior molar tooth, which was decayed and very tender and painful to pressure. I therefore extracted the tooth, and the hard tumor vanished in a few days.

May 8th and 9th. The diseased parts continued to suppurate, and on examination I found more dead bone which required to be removed. I accordingly operated with great care, and extracted a large bone, being a part of the sphenoid or orbitar process, of one inch and three-eighths long, and five-eighths of an inch wide, also several pieces of the nasal plate, the os spongiosa of the right nostril, and the os unguis. The injections of sulphate of zinc and tincture of myrrh, and the emollient poultices continued.

I may here state, as the first incision on the face did not heal more than half up in consequence of its profuse suppuration, it enabled me to remove the latter bones on the 9th of May.

May 10th and 12th.—I found my patient much improved, his eyesight being much strengthened, and relieved of pain, swelling quite reduced. The opening in the alveolus is now quite free, and from which there is a copious flow of pus; and the opening on the cheek has assumed a granulated appearance. For the inflammation of the eye, I prescribed a lotion of sulphate of zinc, acetate of lead, tincture opium and rose water.

May 13th, to the 18th.—The foregoing treatment was continued up to this date, with decided improvement.

May 19th.—To-day I commenced injecting the sinus with diluted port wine, from which the patient did not experience the slightest pain. The discharge of pus is but trifling and the secretions are assuming a normal condition. As my patient is desirous to go to the country for a few days, I prepared for him an astringent lotion for the mouth and throat, and removed from his teeth the tartar which had accumulated in considerable quantity. I also directed the emollient poultices to be continued, and moderate pressure with the bandage to be made over the diseased parts.

May 22d.—My patient returned home after three days absence much improved in every respect—same treatment continued.

May 26th.—On examination, this morning, I observed that the palate bone of the right side remained considerably swollen, and on probing the same through the alveolar puncture with a curved probe, I discovered an abcess about two inches long attached near the cribriformed bone. This, I at length, and with much difficulty, succeeded in extracting, and found it to be shaped like and resembled a cocoon, which, on being opened, was found filled with pus and flocculi. I now injected the parts with the lotion of sulphate of zinc and tincture of myrrh.

May 27th, to the 30th.—My patient absented himself by a visit to the country. He returned home on the 15th of June, and on examination, I found all the disease entirely removed, the puncture in the alveolus remaining open and free from discharge.

July 1st.—To-day I gave the sinus several injections, and finding the parts perfectly recovered, I scarified the gum at the opening in the mouth, and brought the same in contact, and applied the necessary dressing to keep them united.

July 6th.—The incision in the gum I found entirely closed, and all traces of this truly dreadful and extensive disease of the antrum maxillare and adjacent parts are completely removed.

July, 1850.—I have seen my patient several times lately, and he still continues to enjoy perfect health, there never having been the least tendency to a return of the disease.

### ARTICLE VIII.

*Importance of the Preservation of the Teeth.* By L. S. PARMLY, M. D.

